# Pirfenidone treatment in idiopathic pulmonary fibrosis: nationwide Danish results

**DOI:** 10.3402/ecrj.v3.32608

**Published:** 2016-09-09

**Authors:** Goran Nadir Salih, Saher Burhan Shaker, Helle Dall Madsen, Elisabeth Bendstrup

**Affiliations:** 1 Department of Respiratory Medicine, Gentofte University Hospital, Hellerup, Denmark; 2 Department of Respiratory Medicine, Odense University Hospital, Odense, Denmark; 3 Department of Respiratory Diseases and Allergy, Aarhus University Hospital, Aarhus, Denmark

**Keywords:** efficacy, idiopathic pulmonary fibrosis, pirfenidone, safety, therapy

## Abstract

**Background:**

Pirfenidone was approved by the European Medicines Agency and introduced in most European countries in 2011 for treatment of idiopathic pulmonary fibrosis (IPF).

**Objective:**

To describe the national Danish experiences of pirfenidone treatment for IPF during 30 months with respect to target population, safety, adherence to the treatment and effect analysis in a well-characterised IPF population in a real-life setting.

**Methods:**

Retrospective data collection from medical records of all patients in Denmark with IPF from 2011 to 2014. Data included baseline demographics, high-resolution computed tomography (HRCT), histopathology, forced vital capacity (FVC) and 6-min walk test (6MWT). Longitudinal data on FVC, walk test, adherence to the treatment and vital status were also collected.

**Results:**

Pirfenidone treatment was initiated in 113 patients. Mean age was 69.6±8.1 years (±SD), and 71% were male. Definite IPF diagnosis required thoracoscopic lung biopsy in 45 patients (39.8%). The remaining 68 cases had a definite (64 patients) or possible usual interstitial pneumonia (four patients) pattern on HRCT. Patients were followed for 0.1–33.8 months (median 9.4 months). Fifty-one patients (45.2%) needed dose adjustment, 18 (16%) patients discontinued therapy and 13 patients (11.5%) died. The annual mean decline in FVC was 164 ml (SE 33.2). The decline in 6MWT was 18.2 m (SE 11.2). Nausea (44.2%), fatigue (38.9%) and skin reactions (32.7%) were frequent adverse events.

**Conclusion:**

Patients with IPF treated with pirfenidone experienced tolerable adverse events. Patients were maintained on treatment due to a careful follow-up and dose adjustment programme. The annual decline in physiological parameters and mortality rate was comparable to previous randomised controlled trials.

Idiopathic pulmonary fibrosis (IPF) is a specific type of chronic, progressive fibrosing interstitial pneumonia of unknown origin with a dismal prognosis and a median survival of 3–5 years after confirmed diagnosis (1).

Pirfenidone is the first evidence-based treatment for IPF. Pirfenidone was approved by the European Medicines Agency in February 2011 (2) and has been available in Denmark since December 2011. In 2011, the Danish Society of Respiratory Medicine recommended that treatment for IPF was centralised to three tertiary interstitial lung disease (ILD) centres and that pirfenidone treatment was prescribed to patients with a confident diagnosis of IPF and with mild-to-moderate physiological limitation, that is, within the inclusion and exclusion criteria of the CAPACITY trials (3).

Five randomised controlled trials (RCTs) have shown a clinically meaningful effect of pirfenidone on markers of disease progression such as decline in forced vital capacity (FVC), progression-free survival and distance walked in a 6-min walk test (6MWT) (3–6). A Cochrane meta-analysis on the cumulative data of these trials involving 1155 patients showed that pirfenidone reduced the decline of FVC and the risk of disease progression by 30% compared with placebo (7). At week 72, pirfenidone also reduced the proportion of patients with a decline of 50 m or more in the 6-min walk distance (31% relative reduction vs. placebo) and reduced the risk of death or disease progression (26% reduction vs. placebo) (7). Patients participating in RCTs represent a highly selected group of patients fulfilling strict inclusion and exclusion criteria as shown by the high screen failure of 70% in the ASCEND study (6). In a real-life setting, patients with comorbidities such as concomitant emphysema and heart disease are likely to be included and treated contrary to the RCTs. Therefore, it is important to evaluate if the results of evidence-based therapy can be extrapolated to daily clinical practice.

Nine single-centre studies from Europe and Japan including between 40 and 128 patients have previously reported on the use of pirfenidone in a real-life setting. These studies, similar to RCTs, reported either a stabilisation of pulmonary function or a reduction in FVC decline (8–19). Treatment discontinuation due to adverse events ranged from 1 to 19%.

This study describes the nationwide implementation of pirfenidone treatment for patients with IPF in Denmark. The objective of this study was to describe the target IPF population, safety of pirfenidone, adherence to pirfenidone treatment and its effect in real-life clinical practice.

## Material and methods

The study was a national multicentre study collecting retrospective data from electronic patient records at the three Danish tertiary ILD centres at Gentofte University Hospital, Aarhus University Hospital and Odense University Hospital. All patients with a confident diagnosis of IPF according to the 2011 ATS/ERS/JRS/ALAT statement (1) and who had received at least one dose of pirfenidone between December 2011 and 30 September 2014 were included. Nine patients with a definite usual interstitial pneumonia (UIP) pattern on high-resolution computed tomography (HRCT) diagnosed before 2011 had a surgical lung biopsy confirming the diagnosis. The study period was 33 months. Based on the recommendations of the Danish Society of Respiratory Medicine, only patients with mild-to-moderate IPF were considered eligible for pirfenidone treatment; thus, most patients fulfilled the inclusion and exclusion criteria of the CAPACITY trials (3). Initially, patients with diffusing capacity for carbon monoxide (DL_CO_) below 35% of predicted were not considered eligible for pirfenidone treatment. After the publication of the ASCEND trial (6) in May 2014, the threshold for eligibility was, however, changed to DL_CO_ above 30%.

The data collection included baseline demography, HRCT patterns, histopathology, FVC, FVC percent of predicted (FVC%), forced expiratory volume in 1 sec (FEV1), FEV1 percent of predicted (FEV1%), DL_CO_ percent of predicted (DL_CO_%) and the 6MWT. In addition, longitudinal data on lung function, 6MWT, adverse events, adherence to treatment and vital status were collected. All patients had baseline liver function tests and full blood count tests prior to commencing pirfenidone treatment.

Patients were monitored every 3–6 months in their ILD outpatient clinic. All patients had telephone access to a patient responsible nurse. Treatment-related adverse events and treatment compliance were recorded at each visit.

The study protocol was approved by The Danish Data Protection Agency. The local ethics committees needed no approval, as the study was retrospective and pirfenidone was already approved in Denmark.

### Pirfenidone administration

The pirfenidone dose was as a standard escalated over 2 weeks to the full recommended dose of 2,403 mg. In line with recent expert recommendations, dose adjustment measures were undertaken in case of side effects until symptoms resolved (20). Dose adjustment measures included dose reduction, dose interruption, later re-challenging and sometimes a slower re-escalation in accordance with clinical judgment and patient acceptance.

### Patient responsible nurse

Dedicated nurses specialised in IPF and caring for and advising patients in the outpatient setting were available to the patients in all participating ILD centres by telephone. The nurses informed the patients and spouses thoroughly on how to take pirfenidone capsules with food, that the three capsules can be taken separately during the meal and that gastrointestinal side effects typically are expected to be worse in the first 4–6 weeks of treatment. Patients were also educated in avoiding unnecessary exposure to sun and in the proper use of sun screen, and encouraged to avoid outdoor activities in the middle of the day and in the hours immediately after ingestion of capsules. The nurses handed out patient information leaflets and gave advice on other prophylactic means against possible adverse events and management strategies to manage any potential adverse events. Either a visit to the clinic or a telephone consultation with the nurse was scheduled 2–3 weeks after starting pirfenidone treatment. Nurses were allowed to reduce and re-escalate pirfenidone doses and could also guide patients in questions regarding long-term oxygen therapy and ambulatory oxygen therapy. If needed, a pulmonologist specialised in IPF could be consulted. In the case of acute admission, the nurse could be contacted to coordinate follow-up after discharge.

### Statistical analysis

For the description of baseline demographics, data are presented as mean and standard deviation unless otherwise stated. For the longitudinal analysis, all available data were included. A random coefficient regression model with linear time effect was applied. Median survival was estimated using the Kaplan–Meier method. The statistical analysis was performed using the SAS statistical software version 9.2.

## Results

### Baseline demographics

From 21 December 2011 to 30 September 2014, a total of 113 patients with IPF who had received at least one dose of pirfenidone were included. Patients were followed for 0.1–33.8 months (median 9.4 months). The baseline characteristics of the study cohort are summarised in Table 1.

**Table 1:** Baseline characteristics of patients treated with pirfenidone (*n*=113). Figures are mean±SD unless otherwise stated

Characteristics	Value	Range
Age (years)	69.6±8.1	43.0–82.0
Males, *n* (%)	80 (71%)	
Body mass index	26.8±4.0	15.6–37.7
Smoking history% (current/former/never)	0/68/32	
FEV1/FVC	0.80±0.08	0.55–0.95
FVC (*L*)	2.84±0.9	1.24–5.45
FVC% predicted	80.3±17.9	46–121
DL_CO_% predicted	45.9±10.3	18–70
6MWD (*m*)	445.7±94.1	145–640
Oxygen saturation at rest (%)	96.6±2.1	90–100
Desaturation at 6MWT (%)	9.1±6.5	−31–1
Desaturation < 88% at 6MWT (*n*)	41	

DL_CO_: carbon monoxide diffusing capacity, FEV1: forced expiratory volume in 1 sec, FVC: forced vital capacity, SD: standard deviation, 6MWD: 6-min walking distance.

Mean age at pirfenidone treatment start was 69.6 (±8.1 years), and 71% of the patients were males. The majority of patients were former smokers (68%). None of the patients were current smokers. At baseline, the mean FVC was 80% (±17.9) and the mean DL_CO_ was 45.9% (±10.3). Eleven percent of patients were on supplemental oxygen therapy.

**Fig. 1:**
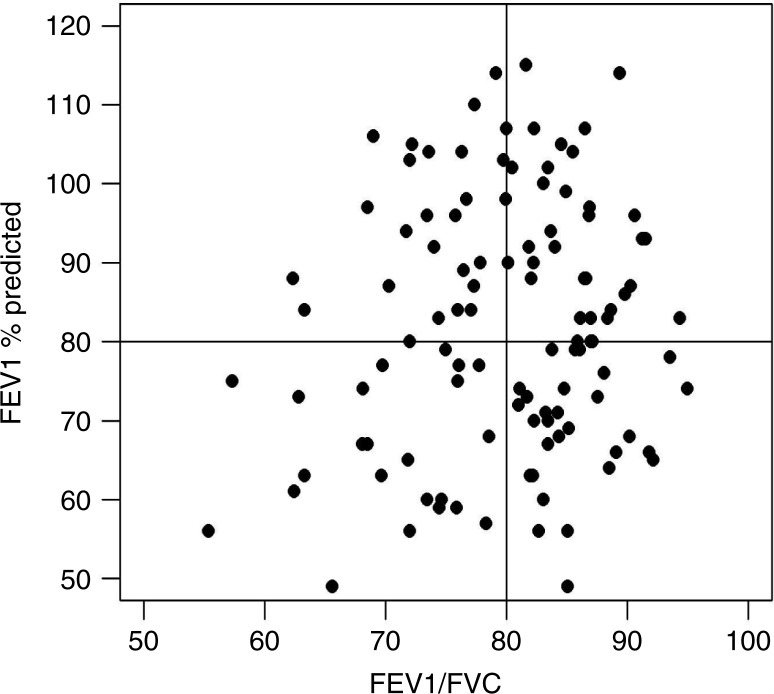
The component of airflow obstruction among the study participants.

Figure 1 shows the distribution of patients with a reduced FEV1/FVC ratio indicative of a mixed obstructive and restrictive decrease in lung function as a function of FEV1. Fourteen (12%) patients had a FEV1/FVC ratio below 0.70.

The diagnosis of IPF was based on clinical evaluation, autoantibody screening (anti-nuclear antibody, anti-neutrophil cytoplasmic antibody, immunoglobulin M rheumatoid factor and anti-citrullinated peptide antibody), various combinations of HRCT and histopathological patterns (Table 2). In 64 patients (56.6%), the diagnosis was based on a definite UIP pattern on HRCT without a surgical lung biopsy. Nine patients with definite UIP pattern on HRCT had undergone a surgical lung biopsy prior to the publication of the current guidelines. In 36 patients (31.9%) with possible or inconsistent HRCT patterns, the diagnosis was confirmed by a surgical lung biopsy and subsequent multidisciplinary conference (MDC) with the participation of pulmonologists, radiologists and pathologists. In four patients (3.5%) with possible UIP pattern on HRCT, clinical information (all four) and bronchoalveolar lavage and transbronchial biopsy findings (three patients) were evaluated at an MDC to obtain a final diagnosis of IPF. Bronchoscopy with bronchoalveolar lavage was performed in 42 patients (37%) and forceps transbronchial biopsies in 23% of patients. However, transbronchial biopsies only contributed to the diagnosis in few patients.

**Table 2:** Diagnosis of IPF

		Video-assisted thoracoscopic lung biopsy			
		
HRCT	Number	Not performed	Definite	Probable	Possible
Definite	73	64	8	1	0
Possible	34	4	20	3	7
Inconsistent	6	0	6	0	0

The annual decline in FVC (mean±standard error of the mean (SEM)) from baseline during 33 months of follow-up was −164.0±33.2 ml (FVC% −3.6%±1.0). The annual decline in DL_CO_% from baseline was −2.2±0.8% (mean±SEM). The decline in 6MWT from baseline was −18.2±11.2 m (mean±SEM).

**Fig. 2:**
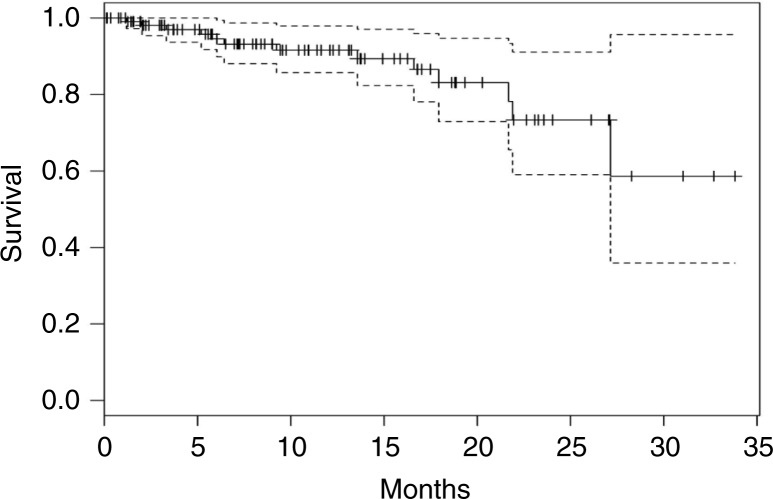
The Kaplan–Meier survival curve with 95% confidence intervals.

Thirteen patients (11.5%) died during the 33 months corresponding to an estimated annual all-cause mortality rate of 14.7%. The Kaplan–Meier plot of the group for all-cause mortality estimate is shown in Fig. 2.

### Safety

Eighteen patients (16%) discontinued pirfenidone treatment due to adverse events. Table 3 summarises adverse events with a frequency of more than 5%. Fifty-one patients (45.2%) needed dose adjustment to continue adherence to treatment.

**Table 3:** Adverse events with a frequency of more than 5%

Adverse events	No.	Percent
Nausea	50	44.2
Vomiting	12	10.6
Diarrhoea	25	22.1
Dyspepsia	35	31.0
Decreased appetite	45	39.8
Weight loss	26	23.0
Taste disturbance	7	6.2
Cough	25	22.1
Dizziness	14	12.4
Fatigue	44	38.9
Insomnia	13	11.5
Skin-related reactions	37	32.7
Arthralgia/muscle pain	10	8.8

Nausea occurred in 44% of patients and fatigue in 38.9%. Skin-related adverse events, primarily photosensitivity, were common and occurred in 32.7%.

The adverse events were mild to moderate and mostly tended to occur within the first 8–10 weeks of treatment. No severe, new or unexpected adverse events were observed.

## Discussion

This study is the first national multicentre study including all patients with IPF and treated with pirfenidone in a real-life setting. The main findings are that pirfenidone treatment in clinical practice is associated with a similar reduction of FVC decline and the same number of adverse events as in the intervention groups in existing RCTs. A careful dose adjustment programme coordinated by physicians and dedicated nurses ensured a high adherence to pirfenidone treatment.

All patients in this study had mild-to-moderate IPF reflected by a mean FVC of 80.3% of predicted, a mean DL_CO_ of 45.9% and a mean 6MWT of 446 m. The mean age was 69 years, and the majority of patients were former smokers and males. All patients met most of the inclusion criteria of the CAPACITY trials and are thus comparable with the patient cohorts from RCTs. However, we included more patients with comorbidities such as emphysema and heart disease as reflected in the number of included patients (14 patients) with a FEV1/FVC ratio below 0.70. In spite of this, the annual rate of decline in FVC was similar to the intervention groups in the CAPACITY and ASCEND studies (3, 6).

The number of diagnosed and treated patients in Denmark is far less than the estimated incidence rates, although regional differences exist. There are a number of possible explanations for this difference. Firstly, a guideline-based confident diagnosis of IPF requires a surgical lung biopsy in patients with a possible UIP pattern on HRCT. It is well known in clinical practice that many patients are not referred to surgical lung biopsy due to procedure risk (comorbidities, high age and low DL_CO_) or patient preferences. Secondly, patients with severe IPF, defined as FVC <50% or DL_CO_ <35% of predicted, were not considered for treatment until the results of the ASCEND study were published where the limit of DL_CO_ was changed to 30%. Thirdly, older patients and patients with severe comorbidities are often not prescribed pirfenidone because of a negative risk–benefit relation. Finally, few patients who were eligible for treatment choose to wait and see without medical treatment after thorough information.

The nature of adverse events in our study was similar to that reported in the ASCEND and CAPACITY studies; neither new, unexpected nor any severe side effects were observed. The adverse events were tolerable and almost exclusively mild to moderate in intensity. The most commonly reported adverse events were gastrointestinal and skin-related events, mostly photosensitivity. The frequency of fatigue in our study was higher (38.9%) than in the CAPACITY (7%) and ASCEND (21%) trials (3, 6). Patients reported an increased degree of fatigue few weeks after treatment initiation. A careful clinical evaluation concluded that symptoms of fatigue were drug related rather than disease related in most patients. A clinically significant elevation of liver enzymes was experienced in only one patient (0.9%), and it was reproducible and fully reversible.

Most of the side effects presented in the first weeks of treatment and were managed by a careful proactive and a tailored dose adjustment programme managed by physicians and dedicated nurses thus ensuring a high rate of adherence. The discontinuation rate was low and similar to the RCTs despite more patients with more comorbidities were included. The comprehensive and thorough information given to patients and coordinated by both doctors and nurses contributed to the high adherence rate of 84% in our study.

Skin-related events were primarily photosensitivity, which was resolvable after protective measures were taken. In the case of allergic rashes or if dose adjustment and protective measures were unsuccessful in ameliorating the symptoms, the patients discontinued the treatment.

Nausea was the most frequent gastrointestinal adverse event, reported by 44% of patients in our study, and a little higher than in the active arms of the two multicentre RCTs, where nausea was reported in 36% of cases (3, 6). It was resolvable in almost all cases with the help of the dose adjustment measures, anti-acid or anti-peristaltic drugs.

It has been shown in both phase III RCTs and animal studies that the anti-fibrotic effect of pirfenidone is dose related and maintaining as high a dose as possible is therefore important for treatment outcome (3–7, 21). The dose adjustment measures and the coordinated efforts such as patient education and motivation by both the pulmonologists specialised in IPF and specialised nurses were considered pivotal to the high adherence to treatment.

The annual decline of FVC and 6MWT in our study was encouraging. The mean annual decline in percent of predicted FVC from baseline was 3.6±1.0% and considerably below the 10% decline regarded as a marker of severe progression. The same was the case with the annual mean decline of DL_CO_ by 2.2±0.8% from the baseline, which is likewise below the clinically significant threshold of 15% (1).

The mean annual decline in 6MWT was 18.2±11.2 m. The minimum clinically important difference of the decline in the walk test in patients with IPF has been reported as 24–45 m or more. The longitudinal variation in the 6MWT has been used to predict the disease status and progression (22, 23) as well as an outcome measure in many clinical trials enrolling subjects with IPF (3, 6).

We acknowledge some limitations in drawing any statistical conclusions from our retrospective observational real-world study both because of the relatively short observation period and the lack of an appropriate control group. To further strengthen our efficacy data, it would have been ideal if we could compare the rate of decline of FVC post-treatment to pre-treatment tests. However, this was impossible as most patients were newly referred and without previous pulmonary function test results.

In conclusion, our findings provide further evidence that pirfenidone treatment of patients with IPF in a real-life setting is safe and generally well tolerated. Gastrointestinal side effects, skin events and fatigue were the most commonly reported adverse events. Adverse events were generally mild to moderate in severity and only led to treatment discontinuation in a minority of patients. Adverse events tended mostly to occur during the first 3 months of treatment and were almost always resolvable. The adherence to the treatment was high due to a careful follow-up and dose adjustment programme. The annual decline in physiological parameters was comparable to the active arms of published RCTs.
